# LED-based multicolor extended resolution transmission fluorescence microscopy

**DOI:** 10.1117/1.JBO.30.4.046501

**Published:** 2025-04-08

**Authors:** Huaiyuan Zhang, Yiting Hu, Xingwei Pu, Shizheng Zhang, Yi He, Kun Chen, Ziji Liu

**Affiliations:** University of Electronic Science and Technology of China, School of Optoelectronic Science and Engineering, Chengdu, China

**Keywords:** light-emitting diode, fluorescence imaging, structured illumination microscopy

## Abstract

**Significance:**

The multiplexing capabilities of fluorescence imaging are enhanced by its exceptional molecular specificity with diverse fluorescent probes, making it a powerful tool for studying complex biological structures, organization, and functions. Recent advances in super-resolution fluorescence microscopy have further revolutionized our ability to explore biology and related fields. However, current multicolor super-resolution fluorescence imaging systems often come with high costs and bulky designs.

**Aim:**

We present a multicolor extended resolution fluorescence imaging system that uses light-emitting diode to simplify the optical path, make the design more compact, and reduce system costs.

**Approach:**

This multicolor extended resolution fluorescence imaging system is based on structured illumination, utilizing a simple diffraction unit positioned between the light source and the sample in a wide-field microscope. Notably, this design could be easily integrated into standard widefield microscopes as a convenient add-on unit, enabling extended resolution imaging.

**Results:**

Our system demonstrates concurrent extended resolved imaging of three-color microsphere beads and successfully showcases multicolor extended resolution fluorescence imaging of biological tissue samples, revealing intricate structural details.

**Conclusions:**

This system provides a structurally simple, cost-effective alternative to traditional microscopes, offering flexible multicolor extended resolution fluorescence imaging and potential applications in multimodal imaging.

## 1 Introduction

Fluorescence microscopy is widely used in biomedical research for its ability to provide multicolor visualization of biological targets with remarkable contrast, making it indispensable for observing complex structures and functions.^1^ However, its spatial resolution is inherently limited by light diffraction. To surpass this limitation, several super-resolution fluorescence microscopy techniques have been developed,^2^^–^^16^ enabling the visualization of subcellular structures and interactions at unprecedented detail. Key methods include stimulated emission depletion microscopy,^2^^–^^6^ single-molecule localization microscopy,^7^^–^^9^ and structured illumination microscopy (SIM).^10^^–^^16^ SIM enhances spatial resolution by shifting high-frequency information from the undetectable region into the detectable range of the Fourier domain through the application of periodic illumination patterns on the sample. As a wide-field imaging technique, SIM is compatible with common fluorescent labels and operates with low illumination power, minimizing the risk of photodamage. These features make SIM particularly well-suited for diverse sample types and experimental conditions, highlighting its potential for various applications in *in vitro* imaging.

Multicolor fluorescence imaging allows for the observation of multiple cellular structures and processes, facilitating the study of cell-protein interactions, dynamic changes, and the interrelationships between these changes and their mutual effects. This provides a more comprehensive understanding of physiological processes and offers a clearer description of disease characteristics.^17^^–^^19^ However, early applications of SIM were limited to imaging single structures in cells.^20^^,^^21^ Gustafsson et al.^22^ advanced the technique using two lasers at distinct wavelengths, enabling concurrent imaging of two different targets. Since then, multicolor SIM systems incorporating multiple lasers and spatial light modulators (SLMs) or digital micromirror devices (DMDs) have been progressively developed.^23^^–^^28^ The combination of lasers with SLM or DMD allows for the generation of high-contrast fringe patterns and rapid modulation, enhancing imaging performance. However, the use of multiple lasers and these modulators introduces significant costs and results in a complex optical path. In addition, as a related issue, the high coherence of lasers inevitably results in speckle noise. Some systems address this issue using a rotating diffuser to reduce the noise, though this adds further complexity to the system.^29^ As a result, despite these technological advancements, current multicolor SIM systems remain costly and complex when multiple color channels are involved.

Although SIM traditionally uses lasers as excitation sources, there has been growing interest in using affordable, incoherent light-emitting diodes (LEDs) for illumination, as they eliminate laser-induced speckle noise and enable microsecond-level output modulation.^30^^,^^31^ Some studies have achieved dual-color SIM imaging using multiple LEDs combined with an SLM.^32^^,^^33^ However, beyond the high cost, pixelated SLMs could introduce unwanted diffraction orders that require masking, and they also necessitate polarization control, further complicating the system. Alternatively, the combination of a DMD and a single LED has been used for dual-color imaging by leveraging the broad absorption spectrum of quantum dots.^34^ However, issues like quantum dot blinking and phototoxicity limit their broader applicability. For super-resolution imaging of tissues or pathological sections, a sufficiently large field of view (FOV) is crucial to capture detailed information across extensive regions of the sample. Although SIM commonly employs SLMs or DMDs to generate structured illumination, the limited number of pixels and size of these devices generally restrict them to producing only a few hundred fringes, which limits the achievable FOV in imaging.

In this work, we present a multicolor SIM system, utilizing a customized programmable multicolor LED array that the color and illumination power of each LED could be controlled individually, a collimator lens and a simple diffraction unit positioned between the light source and the sample in a wide-field microscope. This add-on element is based on a grating and could be easily integrated into existing inverted wide-filed microscope systems, enabling extended resolution (beyond standard resolution achieved by a normal wide-field epifluorescence microscope) imaging. We quantified the lateral extended resolution capability of our system and successfully showcased tri-color extended resolution fluorescence imaging of various regions within a mouse kidney tissue section.

## 2 Methods

### 2.1 Sample Preparation

For beads imaging, polystyrene fluorescent beads (1  μm diameter, excitation/emission wavelengths of 540 nm/580 nm, 1% concentration, Aladdin) were immersed in ethanol to prepare a 1:200 bead–ethanol solution; specifically, 0.05 mL of the beads solution was mixed with 10 mL of ethanol solution. At room temperature, 0.05 mL of this solution was pipetted onto a slide and allowed to solidify.

For biological sample imaging, the paraffin section of mouse kidney tissue was stained with iFluor 488 wheat germ agglutinin (WGA) (excitation: 491 nm; emission: 516 nm), iFluor 546 Vimentin (excitation: 541 nm; emission: 557 nm), and iFluor 647 α-smooth muscle actin (α-SMA) (excitation: 656 nm; emission: 670 nm), purchased from Wuhan Servicebio Technology Co., Ltd., China.

### 2.2 Experiment Setup

**Fig. 1:**
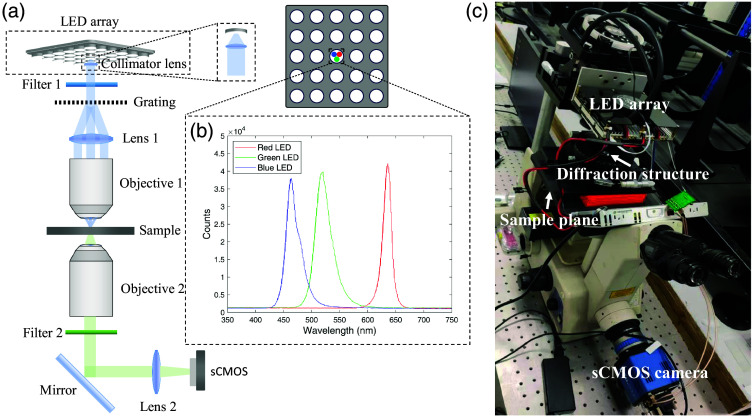
(a) System diagram. The system comprises a multicolor LED array with a collimator lens, a diffraction structure, and an imaging end. The diffraction structure consists of a diffraction grating, a lens, and an objective. It could be easily integrated into existing inverted wide-field microscopy systems. (b) Spectrum of each wavelength module in the LED array, detected using spectrometer (FX2000, ideaoptics). (c) LED array is synchronized with an sCMOS camera to enable rapid switching of LED wavelengths.

The prototype imaging system used in this study is based on an inverted wide-field microscope (TE300, Nikon, Tokyo, Japan). A custom-made, 32×32 programmable multicolor LED array (4-mm spacing), with a collimator lens, was positioned 60 mm above the sample, replacing the original light source on the microscope. The LED illumination angle is ∼120  deg in the spatial circular cone area. We employ a convex lens with a diameter of only 4 mm and a short focal length of 3 mm to collimate the light rays and concentrate the illumination power. A single tri-color LED is used for illumination, and the LED array simplifies the alignment of the light source and optical path. The LED operates in static display mode, with the power of the 465-, 523-, and 632-nm channels being 42, 64, and 64 mW, respectively. The corresponding luminous flux values could be calculated as 1.81, 2.30, and 0.70 lm, respectively. A custom-made diffraction unit was inserted between the light source and the sample to introduce structured illumination, enabling the construction of a simple and compact transmission SIM system, as illustrated in Fig. 1. The diffraction unit consists of a diffraction grating, a convex lens [Lens 1 as shown in Fig. 1(a)] with a 30-mm focal length, and an illuminating objective (20×/0.45 NA, Nikon). The polyethylene terephthalate diffraction grating (JiuZhen Technology Co., Ltd., Shenzhen, China), with a thickness of 0.1 mm and a periodic structure of 100 lines/mm, is responsible for creating periodic structured patterns that are projected onto the sample. The convex lens and illuminating objective create a pair of conjugate planes between the grating and the sample plane. Due to their finite aperture, only the 0th-order and ±1st-order diffraction spots are projected onto the image plane, producing an illumination pattern on the sample with a period of d=3.2  μm. The expected resolution enhancement is, for example, $1+(0.61\;\lambda_{\mathrm{em}}/\mathrm{NA})\cdot (2/d)=1.85$ at an emission wavelength of 670 nm.^14^ This diffraction unit could be seamlessly integrated into existing inverted wide-field microscopy systems. To achieve phase shifting and near-isotropic resolution enhancement, the grating is mounted on a displacement platform (TSD-1202SH, KSPB-1206MH, OptoSigma, Les Ulis, France). The displacement platform is an x-y-θ translation stage. When the mechanical platform laterally displaces and rotates the grating, the periodic pattern shifts spatially. Specifically, each time the grating is mechanically displaced by 1/5 of the spatial period, a phase shift of 2π/5 occurs. After four displacements, a five-step phase shift is obtained in that direction, after which the grating is rotated to the next direction. A five-step phase shift is performed at three angles—0, 60, and 120 deg—using this platform. Fluorescence imaging is captured using a 10×/0.3 NA objective (Nikon) and a single-channel scientific Complementary Metal–Oxide–Semiconductor (sCMOS) camera (PCO.edge 5.5). The raw images obtained were then processed and reconstructed using open-source MATLAB code to produce images with extended resolution.^13^ All image analyses, such as Gaussian fitting of intensity distributions, intensity traces, and resolution estimation, were performed using MATLAB. In addition, all images were further processed with ImageJ, including the application of pseudocolor and channel merging.

We used tri-color enhanced optical power LEDs (LRTB G6TG, OSRAM, Munich, Germany), with a coupling collimator lens that could collimate and collect the majority of the light at central wavelengths of 465, 523, and 632 nm for the three-color channels. Each wavelength has bandwidths of ∼25, 33, and 20 nm, respectively, which provide three partially coherent, quasi-monochromatic light sources. The LED spectrum was measured by a fiber optic spectrometer (FX2000, ideaoptics, Shanghai, China), as shown in Fig. 1(b). Difference from diode lasers, which typically have a full width at half maximum (FWHM) of just 1 pm, LEDs exhibit a broader spectral bandwidth, around 30 nm. This broader bandwidth could be problematic in fluorescence imaging, as the excitation light may overlap with the emission wavelength, increasing background noise. To address this issue, we implemented two bandpass filters—one positioned behind the LED and the other behind the objective lens—to minimize stray light and reduce crosstalk in multicolor fluorescence imaging. To be specific, our system employs excitation and emission filters corresponding to EGFP, mkO, and Cy5 filter sets (Ex/Em: 450 to 490 nm/500 to 550 nm; 515 to 545 nm/555 to 595 nm; 595 to 645 nm/665 to 715 nm), placed behind both the LED array and the objective lens. This configuration, on the other hand, eliminates the need for dichroic mirrors, thereby further reducing the overall system cost.

### 2.3 Reconstruction of SIM Images

In consideration of the structural simplicity, the 0th-order diffracted light was not filtered out, and the optical setup actually generates a three-dimensional (3D) SIM pattern. However, this work focuses solely on two-dimensional imaging, which is equivalent to single-layer 3D SIM. The excitation light emitted from the LED passes through the diffraction unit, forming an illumination pattern on the sample, simplistically expressed in Eq. (1)^35^

$$I(r)=I_{0}[1+m_{1}\;\cos(2\pi pr+\varphi )+m_{2}\;\cos(4\pi pr+2\varphi )],$$ (1)

where $I_{0}$ represents the peak illumination intensity; r is the image spatial coordinates; $m_{1}$ and $m_{2}$ stand for the modulation depth of the first-order and second-order harmonics, respectively; p is the spatial frequency of the illumination; and φ is the phase. The fluorescence emission field on the sample plane is the product of the illumination function and the fluorophore density distribution S of the sample, which could be expressed as Eq. (2)

L=I·S. (2)

By passing through the optical system, the fluorescence emitted from the sample has an emission distribution as shown in Eq. (3)

D=L⊗H=(I·S)⊗H, (3)

where H represents the point spread function of the system, and the symbol ⊗ denotes the convolution operation. Therefore, the spectrum of the original image could be obtained by Eq. (4)^12^

$$\overset{˜}{D}\left(k\right)=[\overset{˜}{I}(k)⊗\overset{˜}{S}(k)]\cdot \overset{˜}{H}(k)=I_{0}[\overset{˜}{S}(k)+\frac{m_{1}}{2}(\overset{˜}{S}(k-p)e^{i\varphi }+\overset{˜}{S}(k+p)e^{-i\varphi })+\frac{m_{2}}{2}(\overset{˜}{S}(k-2p)e^{i2\varphi }+\overset{˜}{S}(k+2p)e^{-i2\varphi })]\cdot \overset{˜}{H}(k),$$ (4)

where k is the spectrum coordinates, $\overset{˜}{\cdot }$ represents the Fourier transform of ·, and the five frequency components of the sample can be separated, shifted, and superimposed in a manner similar to two-dimensional SIM. For each angle of the illumination pattern, five phases should be used; thus, the five equations can be listed in matrix form, as shown in Eq. (5)

$$\left[\begin{array}{c} {\overset{˜}{D}}_{\varphi_{1}}(k)\\ {\overset{˜}{D}}_{\varphi_{2}}(k)\\ {\overset{˜}{D}}_{\varphi_{3}}(k)\\ {\overset{˜}{D}}_{\varphi_{4}}(k)\\ {\overset{˜}{D}}_{\varphi_{5}}(k) \end{array}]=I_{0}M[\begin{array}{c} \overset{˜}{S}(k)\cdot \overset{˜}{H}(k)\\ \overset{˜}{S}(k-p)\cdot \overset{˜}{H}(k)\\ \overset{˜}{S}(k+p)\cdot \overset{˜}{H}(k)\\ \overset{˜}{S}(k-2p)\cdot \overset{˜}{H}(k)\\ \overset{˜}{S}(k+2p)\cdot \overset{˜}{H}(k) \end{array}\right]$$ (5)

where

$$M=[\begin{array}{ccccc} 1 & \frac{m_{1}}{2}e^{i\varphi_{1}} & \frac{m_{1}}{2}e^{-i\varphi_{1}} & \frac{m_{2}}{2}e^{i2\varphi_{1}} & \frac{m_{2}}{2}e^{-i2\varphi_{1}}\\ 1 & \frac{m_{1}}{2}e^{i\varphi_{2}} & \frac{m_{1}}{2}e^{-i\varphi_{2}} & \frac{m_{2}}{2}e^{i2\varphi_{2}} & \frac{m_{2}}{2}e^{-i2\varphi_{2}}\\ 1 & \frac{m_{1}}{2}e^{i\varphi_{3}} & \frac{m_{1}}{2}e^{-i\varphi_{3}} & \frac{m_{2}}{2}e^{i2\varphi_{3}} & \frac{m_{2}}{2}e^{-i2\varphi_{3}}\\ 1 & \frac{m_{1}}{2}e^{i\varphi_{4}} & \frac{m_{1}}{2}e^{-i\varphi_{4}} & \frac{m_{2}}{2}e^{i2\varphi_{4}} & \frac{m_{2}}{2}e^{-i2\varphi_{4}}\\ 1 & \frac{m_{1}}{2}e^{i\varphi_{5}} & \frac{m_{1}}{2}e^{-i\varphi_{5}} & \frac{m_{2}}{2}e^{i2\varphi_{5}} & \frac{m_{2}}{2}e^{-i2\varphi_{5}} \end{array}],$$

so the five frequency components can be expressed as Eq. (6)

$$\left[\begin{array}{c} {\overset{˜}{C}}_{0}(k)\\ {\overset{˜}{C}}_{+1}(k)\\ {\overset{˜}{C}}_{-1}(k)\\ {\overset{˜}{C}}_{+2}(k)\\ {\overset{˜}{C}}_{-2}(k) \end{array}]=[\begin{array}{c} \overset{˜}{S}(k)\cdot \overset{˜}{H}(k)\\ \overset{˜}{S}(k-p)\cdot \overset{˜}{H}(k)\\ \overset{˜}{S}(k+p)\cdot \overset{˜}{H}(k)\\ \overset{˜}{S}(k-2p)\cdot \overset{˜}{H}(k)\\ \overset{˜}{S}(k+2p)\cdot \overset{˜}{H}(k) \end{array}]=\frac{1}{I_{0}}M^{-1}[\begin{array}{c} {\overset{˜}{D}}_{\varphi_{1}}(k)\\ {\overset{˜}{D}}_{\varphi_{2}}(k)\\ {\overset{˜}{D}}_{\varphi_{3}}(k)\\ {\overset{˜}{D}}_{\varphi_{4}}(k)\\ {\overset{˜}{D}}_{\varphi_{5}}(k) \end{array}\right]$$ (6)

where $[\cdot {]}^{-1}$ represents the inverse transformation of the matrix. All spectral components could be resolved by acquiring raw SIM data at different phases. Then, the five frequency components ${\tilde{C}}_{n}(k)$ could be shifted to the correct position, and n represents the index of five frequency components, expressed in Eq. (7)^12^

$${\overset{˜}{C}}_{sn}(k)=F\{F^{-1}[{\overset{˜}{C}}_{n}(k)]\cdot e^{-i2\pi npr}\},$$ (7)

where F and $F^{-1}$ is Fourier transform and inverse Fourier transform, respectively. The frequency domain of the initial extended resolution image ${\tilde{S}}_{\mathrm{er}0}(k)$ is the sum of all the shifted components deconvoluted with ${\tilde{H}}^{*}(k)$ (symbol * denotes the conjugate operation), as shown in Eq. (8)

$${\overset{˜}{S}}_{\mathrm{er}0}(k)=\overset{n=2}{\underset{n=-2}{\sum }}{\overset{˜}{C}}_{sn}(k)\cdot {\overset{˜}{H}}^{*}(k+np),$$ (8)

and the final reconstructed spectrum of SIM images obtained by spectrum optimization developed in Ref. 13.

To accurately separate and shift the spectral components, it is necessary to precisely determine the illumination pattern parameters from the raw SIM data. To do this, the parameters of the fringe pattern, such as spatial frequency and modulation depth, are automatically estimated during the reconstruction process through amplitude-normalized cross-correlation. The above reconstruction has been programmed with MATLAB, which can be freely found in Ref. 13.

## 3 Results

### 3.1 Quantifying the Extended Resolution Capability of the System

**Fig. 2:**
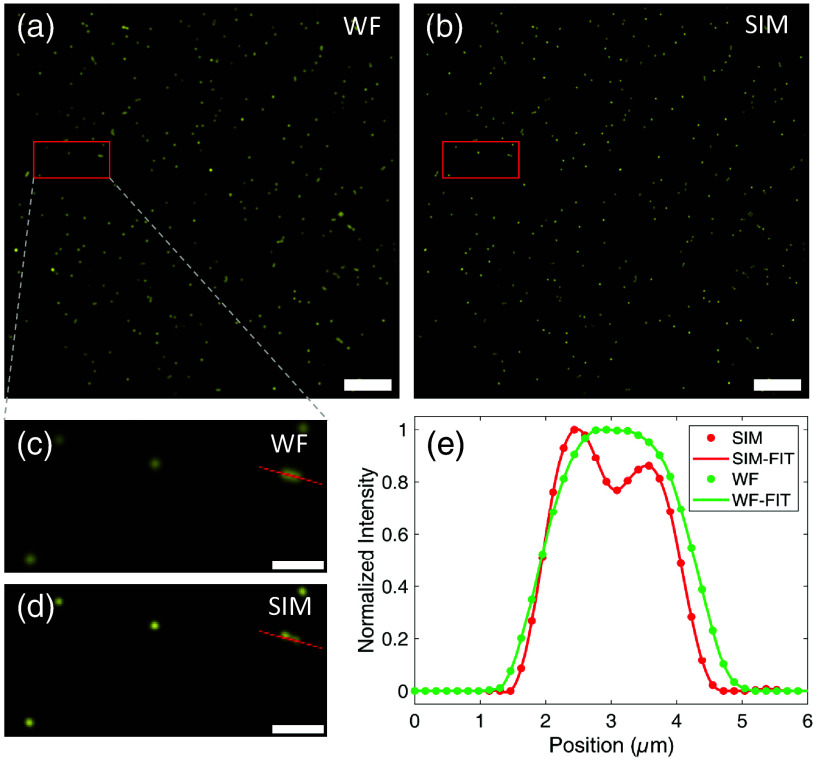
Conventional wide-field imaging and SIM imaging of fluorescence beads. (a) and (b) Wide-field image and SIM image of the beads sample. (c) and (d) Enlarged region of interest (ROI) indicated by the red box in panels (a) and (b). (e) Grayscale distribution along the red line in panels (c) and (d). Scale bars: 20  μm (a) and (b); 5  μm (c) and (d).

To evaluate the extended resolution capability of the imaging system, we employed fluorescent beads in this experiment. Figure 2 shows the comparison results between conventional wide-field and SIM images of the beads. The spatial resolution was measured by averaging spatial measurements of ten 1  μm fluorescent beads. Lines passing through the center of each bead were selected in both the wide-field (WF) and SIM images, and the corresponding intensity distribution curves were obtained. Each intensity distribution curve was fitted with a Gaussian function to determine the FWHM. In the conventional image [Fig. 2(a)], the average FWHM is measured to be 1.24±0.05  μm, which complies with the system’s diffraction limit d=0.61 λ/NA=1.18  μm, determined by the numerical aperture of the objective (10×/0.3 NA, Nikon) and the emission wavelength of the fluorescent beads (580 nm). In contrast, the SIM image [Fig. 2(b)] shows a significant reduction in the average FWHM to 0.83±0.03  μm, which indicates a clear improvement of resolution, beyond the standard resolution of the system. Further, the resolution enhancement is demonstrated in Figs. 2(c) and 2(d), where two adjacent fluorescent beads, indistinguishable in the conventional wide-field image, are clearly resolved in the SIM image.

### 3.2 Imaging Capability for Biological Sample

**Fig. 3:**
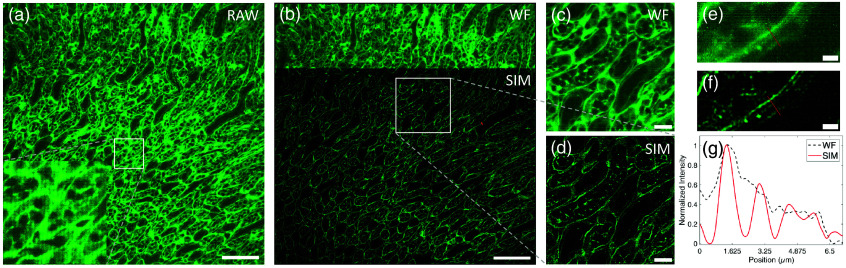
Single-color SIM imaging of ISOM. (a) One of the raw images is shown, and the lower-left illustration is an enlarged view of the region outlined by a white box. (b) Conventional wide-field (top) and SIM (bottom) images reveal significant enhancements in resolution and contrast in the SIM images. (c) and (d) Enlarged ROI, indicated by white boxes in panel (b). The normalized intensity distribution is plotted in panel (g) along the lines shown in panels (e) and (f). The black dashed line represents the wide-field image, whereas the red solid line represents the SIM image. Scale bars: 100  μm (a) and (b); 20  μm (c) and (d); 5  μm (e) and (f).

We also demonstrated the capability of our system by imaging of mouse kidney tissue section. In the 465-nm channel, the inner stripe of the outer medulla (ISOM) is observed, with wheat germ agglutinin conjugated to iFluor 488 serving as a specific marker for tubular epithelial cells. As illustrated in Fig. 3, the SIM images reveal a substantial improvement in resolution compared with the conventional ones, making it possible to distinguish features that are otherwise indiscernible. In the transverse section of the outer medulla, three key structures could be clearly observed: the thin limbs of Henle’s loop, the thick ascending limbs of Henle’s loop (TALs), and the collecting ducts (CDs). The thin limbs are distinguishable from the TAL by their shorter cell height. Furthermore, the TAL’s apical surface is characterized by brush-like microvilli, which are involved in reabsorption processes, whereas the apical surface of the collecting ducts displays fine filaments. These microstructures, difficult to resolve in conventional wide-field images, are rendered sharply in the SIM images, underscoring the enhanced resolution provided by our system. This ability to resolve fine structural details within tissue sections highlights the potential of our system for extended resolution biological imaging, particularly in complex tissue environments.

### 3.3 Multicolor Extended Resolution Imaging Capability

**Fig. 4:**
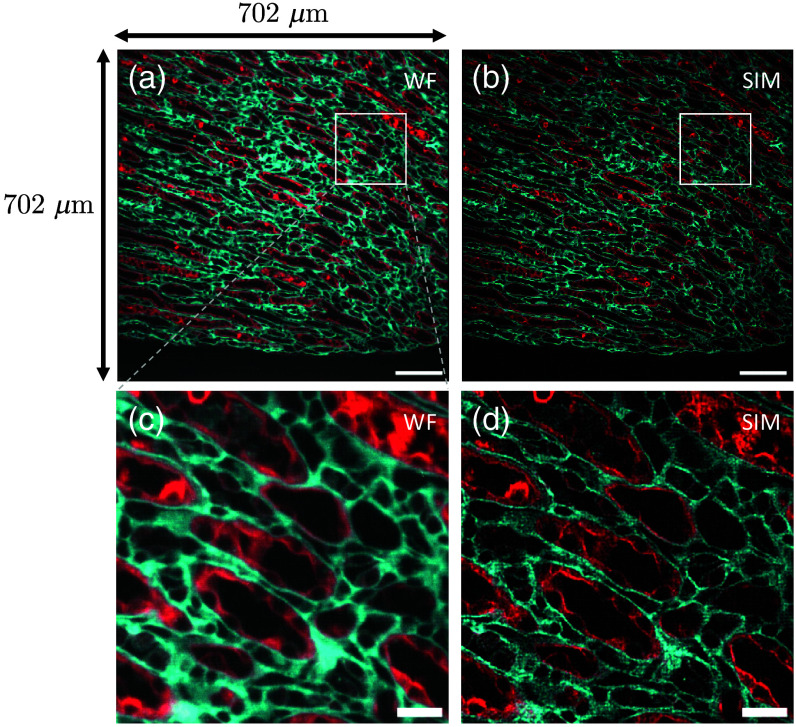
Two-color imaging of the IM. (a) and (b) Conventional wide-field image and SIM image, respectively. (c) and (d) Enlarged ROI indicated by white boxes in panels (a) and (b). α-SMA is used as a marker for myofibroblasts. Scale bars: 100  μm (a) and (b); 20  μm (c) and (d).

**Fig. 5:**
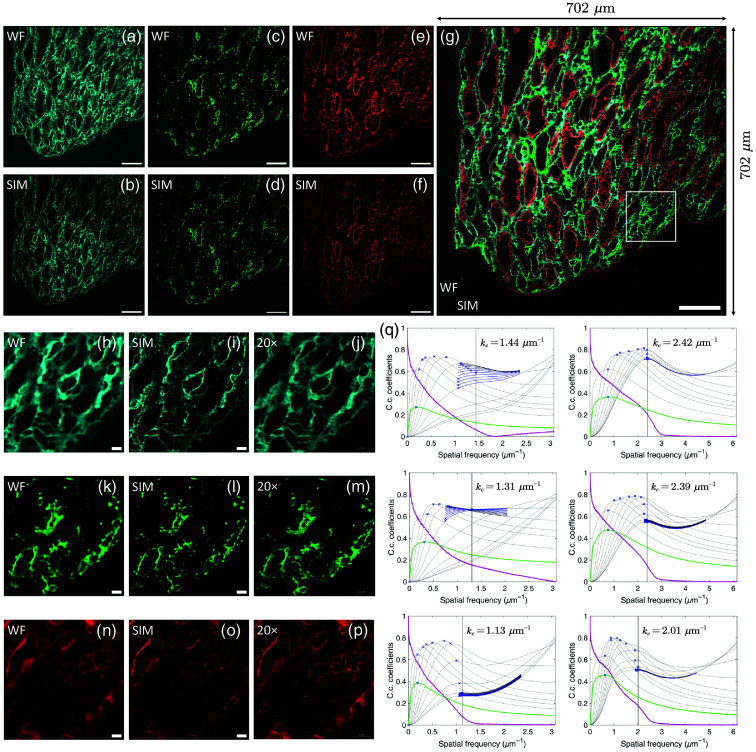
Three-color imaging of renal papilla. (a), (c), and (e) Conventional wide-field images. (b), (d), and (f) SIM images, with tubular epithelial cells in the 465 nm channel (cyan), mesenchymal cells in the 523-nm channel (green), and myofibroblasts in the 632-nm channel (red). (g) Overlay of all three channels, with wide-field images in the upper left and SIM images in the lower right. (h)–(p) Enlarged ROI corresponding to the white box in panel (g), with wide-field images (h), (k), and (n) on the left and SIM images (i), (l), and (o) right. (j), (m), and (p) Images captured by the Nikon Eclipse C1 and 3DHISTECH Pannoramic MIDI (20×/0.40 NA), provided by Wuhan Servicebio Technology Co., Ltd, China. (q) Decorrelation analysis of conventional wide-field and SIM images.^36^ Green line, decorrelation functions before high-pass filtering. Magenta line, radial average of the log of the absolute value of Fourier transform. Gray lines, all high-pass filtered decorrelation functions. Blue to black lines, decorrelation functions with refined mask radius and high-pass filtering range. Blue crosses, all local maxima. Dashed vertical line, cutoff frequency. From top to bottom are the 465-nm channel, 523-nm channel, and 632-nm channel, with the left row corresponding to WF images and the right row corresponding to SIM images. Scale bars: 100  μm (a)–(g); 10  μm (h)–(p).

In the multicolor SIM imaging, we obtained a two-color image of the inner medulla (IM) and a tri-color image of the renal papilla in mouse kidney tissue section, as shown in Figs. 4 and 5, respectively. The SIM images demonstrate a remarkable improvement in both resolution and contrast across all channels, allowing previously blurred or indistinct filaments to become clearly visible.

In the renal papilla region, co-labeling with different markers reveals distinct spatial distribution patterns, highlighting various cell types, as shown in Fig. 5. WGA staining specifically marks the epithelial cells of the CDs, outlining their lumen or cell membranes. Vimentin labels mesenchymal cells, which are in the renal interstitial area surrounding the CDs. Although conventional wide-field imaging provides a general view of the extracellular matrix structure, the finer details of the fibrous structures require the enhanced resolution provided by SIM. α-SMA stains myofibroblasts, which are positioned around the blood vessels and CDs, forming either circular or parallel arrangements along the WGA-labeled CDs. These myofibroblasts play a crucial role in providing mechanical support, ensuring the structural integrity of the lumen. In SIM images, the arrangement of actin filaments is resolved with much greater detail, whereas conventional images only present their general distribution.

To quantitatively evaluate the resolution enhancement achieved by SIM, we applied decorrelation analysis to analyze the conventional wide-field and SIM images. Decorrelation analysis provides an objective measure of image resolution by utilizing partial phase correlation, making it a robust tool for the evaluation of the improvements in resolution.^36^ The improvement factors are the ratio of the cut-off frequencies of the SIM images to those of the WF images. Accordingly, the resolution enhancement results for the SIM images in the 465-, 523-, and 632-nm channels showed improvements of 1.68, 1.82, and 1.78, respectively. The resolution improvement across the three channels varies slightly and is not monotonic, which is most likely due to differences in the signal-to-noise ratio.

## 4 Discussion

The imaging system proposed in this paper presents an economical and efficient solution for multicolor extended resolution fluorescence microscopy by integrating a programmable multicolor LED array coupling a collimator lens and a simple diffraction structure into an inverted wide-field microscope. The enhancement in lateral resolution was quantified through imaging of fluorescence beads, and the system successfully demonstrated multicolor extended resolution fluorescence imaging across various regions of mouse kidney tissue section, validating its capability for biological tissue imaging.

LEDs offer several advantages for fluorescence microscopy, including discrete and narrow excitation peaks within the visible light spectrum, brightness control, a long lifespan, high stability, and reduced heat generation. As a result, they have become a popular choice for this kind of application. The programmable LED array facilitates rapid wavelength switching and could be seamlessly integrated into existing microscope systems, allowing for the addition of extra LED modules to expand the available wavelength options and functions.

The FOV of the system is determined by the camera’s resolution and the optical magnification. Utilizing the central area (2160×2160  pixels) of the camera (pixel size: 6.5  μm) with an objective magnification of 10×, the effective field of view could span ∼1404  μm×1404  μm (the final FOV is only 702  μm×702  μm due to the prototype microscope system, which includes an intermediate 2× magnification). In contrast, standard SLM and DMD devices, under the same fringe period and five-step phase shift, are only capable of covering a smaller field of view of 1228.8  μm×691.2  μm, due to their typical resolution of 1920×1080  pixels.

Our developed diffraction structure, based on a diffraction grating, could be readily incorporated into the transmitted optical path of existing inverted microscopes while maintaining high optical efficiency. In addition, it holds promise for multimodal imaging applications. However, a notable drawback of this system is the need to mechanically translate and rotate the grating to acquire raw data for structured illumination, which results in low temporal resolution. To address this, phase shifts could potentially be achieved through electronic switching of the LED array.

Despite this limitation, the proposed multicolor fluorescence imaging system has been shown to deliver extended resolution multicolor fluorescence images while maintaining a simple, compact structure at a high cost-effectiveness. This method enhances the flexibility and scalability of multicolor fluorescence imaging across various application scenarios and offers an affordable and easy-to-implement solution to expand the border range of researchers. Furthermore, this system could be easily extended in different shapes for multimode imaging, allowing for the integration of bright field, dark field,^37^ phase imaging,^38^^,^^39^ and even 3D imaging in both structure and function microscopy.

## 5 Conclusions

In this paper, we present a multicolor extended resolution transmission fluorescence imaging system that employs a programmable multicolor LED array. By replacing the light source of an inverted wide-field microscope and incorporating a simple diffraction structure between the light source and the sample, we successfully obtained multicolor extended resolution fluorescence images of various regions within the mouse kidney tissue section. This hardware part serves as a cost-effective alternative to traditional multicolor extended resolution fluorescence imaging methods and the programmable and flexible illumination pattern and wavelength choice of the LED array allows for adaptation to a diverse range of samples and experimental conditions, which significantly enhances the flexibility and scalability of multicolor fluorescence imaging.

## Data Availability

Code and data underlying the results presented in this paper are not publicly available at this time but may be obtained from the authors upon reasonable request.

## References

[1] Vogler N. et al., “Multimodal imaging spectroscopy of tissue,” Ann. Rev. Anal. Chem. 8(1), 359–387 (2015). 10.1146/annurev-anchem-071114-040352 26070717

[2] Hell S. Wichmann J., “Breaking the diffraction resolution limit by stimulated emission: stimulated-emission depletion fluorescence microscopy,” Opt. Lett. 19(11), 780–782 (1994). OPLEDP 0146-9592 10.1364/ol.19.000780 19844443

[r3] Liu Y. et al., “Amplified stimulated emission in upconversion nanoparticles for super-resolution nanoscopy,” Nature 543(7644), 229–233 (2017). 10.1038/nature21366 28225761

[r4] Wang L. et al., “Low-power STED nanoscopy based on temporal and spatial modulation,” Nano Res. 15(4), 3479–3486 (2021). 10.1007/s12274-021-3874-1

[r5] Weber M. et al., “MINSTED fluorescence localization and nanoscopy,” Nat. Photonics 15(5), 361–366 (2021). NPAHBY 1749-4885 10.1038/s41566-021-00774-2 33953795 PMC7610723

[r6] Tu S.-J. et al., “Shaping the illumination beams for STED imaging through highly scattering media,” Appl. Phys. Lett. 119(21), 211105 (2021). APPLAB 0003-6951 10.1063/5.0066331

[r7] Betzig E. et al., “Imaging intracellular fluorescent proteins at nanometer resolution,” Science 313(5793), 1642–1645 (2006). SCIEAS 0036-8075 10.1126/science.1127344 16902090

[r8] Xie L. et al., “3D ATAC-PALM: super-resolution imaging of the accessible genome,” Nat. Methods 17(4), 430–436 (2020).1548-7091 10.1038/s41592-020-0775-2 32203384 PMC7207063

[r9] Tehrani K. F. et al., “Adaptive optics stochastic optical reconstruction microscopy (AO-STORM) by particle swarm optimization,” Biomed. Opt. Express 8(11), 5087 (2017). BOEICL 2156-7085 10.1364/BOE.8.005087 29188105 PMC5695955

[r10] Gustafsson M. G. L., “Surpassing the lateral resolution limit by a factor of two using structured illumination microscopy,” J. Microsc. 198(2), 82–87 (2001). JMICAR 0022-2720 10.1046/j.1365-2818.2000.00710.x 10810003

[r11] Müller M. et al., “Open-source image reconstruction of super-resolution structured illumination microscopy data in ImageJ,” Nat. Commun. 7(1), 10980 (2016). NCAOBW 2041-1723 10.1038/ncomms10980 26996201 PMC4802170

[r12] Cao R. et al., “Open-3DSIM: an open-source three-dimensional structured illumination microscopy reconstruction platform,” Nat. Methods 20(8), 1183–1186 (2023).1548-7091 10.1038/s41592-023-01958-0 37474809 PMC10406603

[r13] Wen G. et al., “High-fidelity structured illumination microscopy by point-spread-function engineering,” Light, Sci. Appl. 10(1), 70 (2021). 10.1038/s41377-021-00513-w 33795640 PMC8016956

[r14] Zheng J. et al., “Large-field lattice structured illumination microscopy,” Opt. Express 30(15), 27951 (2022). OPEXFF 1094-4087 10.1364/OE.461615 36236953

[r15] Lal A. Shan C. Xi P., “Structured illumination microscopy image reconstruction algorithm,” IEEE J. Sel. Top. Quantum Electron. 22(4), 50–63 (2016). IJSQEN 1077-260X 10.1109/JSTQE.2016.2521542

[16] Liu G. et al., “Miniaturized structured illumination microscopy with diffractive optics,” Photonics Res. 10(5), 1317 (2022). 10.1364/PRJ.450799

[17] Meleppat R. K. et al., “In situ morphologic and spectral characterization of retinal pigment epithelium organelles in mice using multicolor confocal fluorescence imaging,” Investig. Opthalmol. Vis. Sci. 61(13), 1 (2020). 10.1167/iovs.61.13.1 PMC7645167 33137194

[r18] Ramirez M. et al., “3D evaluation of the extracellular matrix of hypoxic pancreatic islets using light sheet fluorescence microscopy,” Islets 16(1), 2298518 (2024). 10.1080/19382014.2023.2298518 38267218 PMC10810165

[19] Long D. et al., “Analysis of Kif5b expression during mouse kidney development,” PloS One 10(4), e0126002 (2015). POLNCL 1932-6203 10.1371/journal.pone.0126002 25885434 PMC4401754

[20] Kner P. et al., “Super-resolution video microscopy of live cells by structured illumination,” Nat. Methods 6(5), 339–342 (2009).1548-7091 10.1038/nmeth.1324 19404253 PMC2895555

[21] Hirvonen L. M. et al., “Structured illumination microscopy of a living cell,” Eur. Biophys. J. 38(6), 807–812 (2009). EBJOE8 0175-7571 10.1007/s00249-009-0501-6 19536533

[22] Gustafsson M. G. L. et al., “Three-dimensional resolution doubling in wide-field fluorescence microscopy by structured illumination,” Biophys. J. 94(12), 4957–4970 (2008). BIOJAU 0006-3495 10.1529/biophysj.107.120345 18326650 PMC2397368

[23] Young L. J. Strohl F. Kaminski C. F., “A guide to structured illumination TIRF microscopy at high speed with multiple colors,” J. Vis. Exp. (111), e53988 (2016). 10.3791/53988 PMC4927749 27285848

[r24] Markwirth A. et al., “Video-rate multi-color structured illumination microscopy with simultaneous real-time reconstruction,” Nat. Commun. 10(1), 4315 (2019). NCAOBW 2041-1723 10.1038/s41467-019-12165-x 31541134 PMC6754501

[r25] Huang X. et al., “Fast, long-term, super-resolution imaging with Hessian structured illumination microscopy,” Nat. Biotechnol. 36(5), 451–459 (2018). NABIF9 1087-0156 10.1038/nbt.4115 29644998

[r26] Zhao T. et al., “Multi-color structured illumination microscopy for live cell imaging based on the enhanced image recombination transform algorithm,” Biomed. Opt. Express 12(6), 3474 (2021). BOEICL 2156-7085 10.1364/BOE.423171 34221673 PMC8221967

[r27] York A. G. et al., “Resolution doubling in live, multicellular organisms via multifocal structured illumination microscopy,” Nat. Methods 9(7), 749–754 (2012).1548-7091 10.1038/nmeth.2025 22581372 PMC3462167

[28] Brown P. T. et al., “Multicolor structured illumination microscopy and quantitative control of polychromatic light with a digital micromirror device,” Biomed. Opt. Express 12(6), 3700 (2021). BOEICL 2156-7085 10.1364/BOE.422703 34221689 PMC8221958

[29] Wen K. et al., “Structured illumination microscopy with partially coherent illumination for phase and fluorescent imaging,” Opt. Express 29(21), 33679 (2021). OPEXFF 1094-4087 10.1364/OE.435783 34809175

[30] Dan D. et al., “DMD-based LED-illumination super-resolution and optical sectioning microscopy,” Sci. Rep. 3(1), 1116 (2013). 10.1038/srep01116 23346373 PMC3552285

[31] Mayerich D. et al., “An LED-based structured illumination microscope using a digital micromirror device and GPU accelerated image reconstruction,” PloS One 17(9), e0273990 (2022). POLNCL 1932-6203 10.1371/journal.pone.0273990 36084054 PMC9462783

[32] Lukeš T. et al., “Three-dimensional super-resolution structured illumination microscopy with maximum a posteriori probability image estimation,” Opt. Express 22(24), 29805 (2014). OPEXFF 1094-4087 10.1364/OE.22.029805 25606910

[33] Pospíšil J. et al., “Imaging tissues and cells beyond the diffraction limit with structured illumination microscopy and Bayesian image reconstruction,” GigaScience 8(1), giy126 (2019). 10.1093/gigascience/giy126 30351383 PMC6325271

[34] Zeng H. et al., “Dual-color quantum dot structured illumination microscopy using single LED,” Proc. SPIE 11337, 113370T (2019). PSISDG 0277-786X 10.1117/12.2545456

[35] Zhao T. et al., “Fast single-layer reconstruction for three-dimensional structured illumination microscopy,” Opt. Lasers Eng. 167, 107606 (2023). OLENDN 0143-8166 10.1016/j.optlaseng.2023.107606

[36] Descloux A. Grußmayer K. S. Radenovic A., “Parameter-free image resolution estimation based on decorrelation analysis,” Nat. Methods 16(9), 918–924 (2019).1548-7091 10.1038/s41592-019-0515-7 31451766

[37] Liu Z. et al., “Real-time brightfield, darkfield, and phase contrast imaging in a light-emitting diode array microscope,” J. Biomed. Opt. 19(10), 106002 (2014). JBOPFO 1083-3668 10.1117/1.JBO.19.10.106002 25271540

[38] Tian L. Waller L., “Quantitative differential phase contrast imaging in an LED array microscope,” Opt. Express 23(9), 11394 (2015). OPEXFF 1094-4087 10.1364/OE.23.011394 25969234

[39] Tian L. et al., “Computational illumination for high-speed in vitro Fourier ptychographic microscopy,” Optica 2(10), 904 (2015). 10.1364/OPTICA.2.000904

